# Comparative Genomic Analysis of Rapidly Evolving SARS-CoV-2 Reveals Mosaic Pattern of Phylogeographical Distribution

**DOI:** 10.1128/mSystems.00505-20

**Published:** 2020-07-28

**Authors:** Roshan Kumar, Helianthous Verma, Nirjara Singhvi, Utkarsh Sood, Vipin Gupta, Mona Singh, Rashmi Kumari, Princy Hira, Shekhar Nagar, Chandni Talwar, Namita Nayyar, Shailly Anand, Charu Dogra Rawat, Mansi Verma, Ram Krishan Negi, Yogendra Singh, Rup Lal

**Affiliations:** a P.G. Department of Zoology, Magadh University, Bodh Gaya, Bihar, India; b Department of Zoology, Ramjas College, University of Delhi, Delhi, India; c Department of Zoology, University of Delhi, Delhi, India; d The Energy and Resources Institute, New Delhi, India; e PhiXGen Private Limited, Gurugram, Haryana, India; f Department of Zoology, College of Commerce, Arts & Science, Patliputra University, Patna, Bihar, India; g Department of Zoology, Maitreyi College, University of Delhi, New Delhi, India; h Department of Zoology, Sri Venkateswara College, University of Delhi, New Delhi, India; i Department of Zoology, Deen Dayal Upadhyaya College, University of Delhi, New Delhi, India; Princeton University

**Keywords:** COVID-2019, SARS-CoV-2, viruses

## Abstract

The outbreak of coronavirus disease 2019 (COVID-19) that started in Wuhan, China, in December 2019 has spread worldwide, emerging as a global pandemic. The severe respiratory pneumonia caused by novel severe acute respiratory syndrome coronavirus 2 (SARS-CoV-2) has so far claimed more than 0.38 million lives and has impacted human lives worldwide. However, as the novel SARS-CoV-2 virus displays high transmission rates, the underlying genomic severity is required to be fully understood. We studied the complete genomes of 95 SARS-CoV-2 strains from different geographical regions worldwide to uncover the pattern of the spread of the virus. We show that there is no direct transmission pattern of the virus among neighboring countries, suggesting that its spread is a result of travel of infected humans to different countries. We revealed unique single nucleotide polymorphisms (SNPs) in nonstructural protein 13 (nsp13), nsp14, nsp15, and nsp16 (ORF1b polyproteins) and in the S-protein within 10 viral isolates from the United States. These viral proteins are involved in RNA replication and binding with the human receptors, indicating that the viral variants that are circulating in the population of the United States are different from those circulating in the populations of other countries. In addition, we found an amino acid addition in nsp16 (mRNA cap-1 methyltransferase) of a U.S. isolate (GenBank accession no. MT188341.1) leading to a shift in the amino acid frame from position 2540 onward. Through comparative structural analysis of the wild-type and mutant proteins, we showed that this addition of a phenylalanine residue renders the protein in the mutant less stable, which might affect mRNA cap-1 methyltransferase function. We further analyzed the SARS-CoV-2–human interactome, which revealed that the interferon signaling pathway is targeted by orf1ab during infection and that it also interacts with NF-κB-repressing factor (NKRF), which is a potential regulator of interleukin-8 (IL-8). We propose that targeting this interaction may subsequently improve the health condition of COVID-19 patients. Our analysis also emphasized that SARS-CoV-2 manipulates spliceosome machinery during infection; hence, targeting splicing might affect viral replication. In conclusion, the replicative machinery of SARS-CoV-2 is targeting interferon and the notch signaling pathway along with spliceosome machinery to evade host challenges.

**IMPORTANCE** The COVID-19 pandemic continues to storm the world, with over 6.5 million cases worldwide. The severity of the disease varies with the territories and is mainly influenced by population density and age factor. In this study, we analyzed the transmission pattern of 95 SARS-CoV-2 genomes isolated from 11 different countries. Our study also revealed several nonsynonymous mutations in ORF1b and S-proteins and the impact on their structural stability. Our analysis showed the manipulation of host system by viral proteins through SARS-CoV-2–human protein interactome, which can be useful to understand the impact of virus on human health.

## Abstract (precis)

The COVID-19 pandemic continues to storm the world, with over 6.5 million cases worldwide. The severity of the disease varies with the territories and is mainly influenced by population density and age factor. In this study, we analyzed the transmission pattern of 95 SARS-CoV-2 genomes isolated from 11 different countries. Our study also revealed several nonsynonymous mutations in ORF1b and S-proteins and the impact on their structural stability. Our analysis showed the manipulation of host system by viral proteins through SARS-CoV-2–human protein interactome, which can be useful to understand the impact of virus on human health.

## INTRODUCTION

Since the current outbreak of pandemic coronavirus disease 2019 (COVID-19) caused by severe acute respiratory syndrome coronavirus 2 (SARS-CoV-2), assessment of the biogeographical pattern of SARS-CoV-2 isolates and the mutations present at the nucleotide and protein levels is of high interest to many research groups (1–3). Coronaviruses (CoVs), members of *Coronaviridae* family, order *Nidovirales*, have been known to be human pathogens for the last 6 decades (4). Their targets are not limited just to humans but also extend to other mammals and birds (5). Coronaviruses have been classified in the alphacoronavirus, betacoronavirus, gammacoronavirus, and deltacoronavirus groups (6), among which the members of the first two groups are known to infect mammals whereas those of the latter two primarily infect bird species (7). Symptoms in humans range from common cold to respiratory and gastrointestinal distress of various intensities. In the past, more-severe forms caused major outbreaks that included infections by severe acute respiratory syndrome coronavirus (SARS-CoV) (outbreak in 2003 in China) and Middle East respiratory syndrome coronavirus (MERS-CoV) (outbreak in 2012 in the Middle East) (8). Among mammals, bats have been shown to host coronaviruses, acting as their natural reservoirs, through which the viruses may be transmitted to humans via an intermediate host (9). However, SARS-CoV and MERS-CoV were found to have been transmitted from the intermediate hosts palm civets and camel, respectively (10, 11). Though it is not yet clear which animal served as the intermediate host for transmission of SARS-CoV-2 from bats to humans, it was suggested previously that it was most likely a warm-blooded vertebrate (12, 13).

The inherently high recombination frequency and mutation rates of coronavirus genomes enable their easy transmission among different hosts. Structurally, they are positive-sense single-stranded RNA (ssRNA) virions with characteristic spikes projecting from the surface of the capsid coating (14, 15). The spherical capsid and spikes give them a crown-like appearance, due to which the viruses were named “corona,” meaning “crown” or “halo” in Latin. Their genome is nearly 30 kb in length, largest among the RNA viruses, with a 5′ cap and a 3′ poly(A) tail, for translation (16). Coronaviruses consist of four main proteins, the spike (S), membrane (M), envelope (E), and nucleocapsid (N) proteins. The spike protein (∼150 kDa) mediates its attachment to host receptor proteins (17). The membrane protein (∼25 to 30 kDa) attaches with nucleocapsid and maintains the curvature of the virus membrane (18). The envelope protein (8 to 12 kDa) is responsible for the pathogenesis of the virus as it eases the assembly and release of virion particles and also has ion channel activity as an integral membrane protein (19). Nucleocapsid, the fourth protein, helps in the packaging of virus particles into capsids and promotes formation of the replicase-transcriptase complex (RTC) (20).

The outbreak of novel betacoronavirus (2019-nCoV), or SARS-CoV-2, in December 2019 in Wuhan, China, has shown devastating effects worldwide. It has emerged as a reason for concern not only for its pneumonia-like symptoms but also for its asymptomatic nature and the challenges encountered in efforts to contain it (21, 22). The World Health Organization (WHO) declared COVID-19 a pandemic on 11 March 2020; however, by the time it was declared a pandemic, more than 118,000 cases of the coronavirus illness had been reported from over 113 countries and territories around the world (WHO Situation Report 51). So far, it has affected more than 200 countries and territories, and the number of deaths resulting from the disease have exceeded 0.38 million worldwide. Virtually all human lives have been impacted, with no foreseeable end of the pandemic. SARS-CoV-2 is assumed to have originated from bats, which serve as a reservoir host of the virus (9). Similar mutation patterns in Bat-SARS-CoV RaTG13 and SARS CoV-2 were also recently revealed, but the data set was limited to 21 strains, including a few SARS-CoV-2 strains and neighboring strains (9). Numerous studies have now reported the genome composition and divergence patterns of SARS-CoV-2 (3, 23). In this study, we selected 95 strains of SARS-CoV-2, isolated from 11 different countries, to understand the transmission patterns, evolution, and pathogenesis of the virus. Using core-genome-based and single nucleotide polymorphism (SNP)-based phylogeny, we attempted to uncover the transmission pattern of the virus across the affected countries, which was not known earlier. We analyzed the open reading frames (ORFs) of the isolates to reveal unique point mutations and amino acid substitutions/additions in the isolates from the United States. In addition, we analyzed the gene/protein mutations in these novel strains and estimated the direction of selection to decipher their evolutionary divergence rate. Further, we also established the interactome of SARS-CoV-2 with the human host proteins to predict the functional implications represented by the viral infected host cells. The results obtained from the analyses indicate different variants of SARS-CoV-2 isolates, with an inherent capability of unique mutations and evolving viral replication system enabling adaptation to human hosts. To our knowledge, this is the first study to demonstrate the biogeographical distribution pattern of this emerging pathogen coupled with the high rate of mutations.

## RESULTS AND DISCUSSION

### General genomic attributes of SARS-CoV-2.

In this study, we analyzed a total of 95 SARS-CoV-2 strains (available on 19 March 2020) isolated and sequenced between December 2019 and March 2020 from 11 different countries, namely, the United States (*n* = 52 isolates), China (*n* = 30), Japan (*n* = 3), India (*n* = 2), Taiwan (*n* = 2), Australia (*n* = 1), Brazil (*n* = 1), Italy (*n* = 1), Nepal (*n* = 1), South Korea (*n* = 1), and Sweden (*n* = 1). A total of 68 strains were isolated from either oronasopharynges or lungs, while two of them were isolated from feces, suggesting both respiratory and gastrointestinal SARS-CoV-2 connections (Table 1). No information concerning the source of isolation of the remaining isolates was available. The average genome size and GC content were found to be 29,879 ± 26.6 bp and 37.99% ± 0.018%, respectively. All these isolates were found to harbor 9 open reading frames (ORFs) coding for ORF1a (13,218-bp) and ORF1b (7,788-bp) polyproteins, surface glycoprotein or S-protein (3,822 bp), ORF3a protein (828 bp), membrane glycoprotein (M-protein) (669 bp), ORF6 protein (186 bp), ORF7a protein (366 bp), ORF8 protein (366 bp), and nucleocapsid phosphoprotein (N-protein) (1,260 bp), which agrees with a recently published study (24). ORF1a harbors 12 nonstructural proteins (nsp), namely, nsp1, nsp2, nsp3 (papain-like protease or PLpro domain), nsp4, nsp5 (3C-like protease [3CLpro]), nsp6, nsp7, nsp8, nsp9, nsp10, nsp11, and nsp12 (RNA-dependent RNA polymerase [RdRp]) (24). Similarly, ORF1b contains four putative nonstructural proteins, namely, nsp13 (helicase or Hel), nsp14 (3′-to-5′ exoribonuclease or ExoN), nsp15, and nsp16 (mRNA cap-1 methyltransferase).

**TABLE 1:** General genomic attributes of SARS-CoV-2 strains

Strain no.	Accession no.	Virus (SARS-CoV-2)	Country of origin	Genome size (bp)	GC%	Isolation source(s)	Date of isolation
1	LC528232.1	Hu/DP/Kng/19-020	Japan	29,902	37.98	Oronasopharynx	10 February 2020
2	LC528233.1	Hu/DP/Kng/19-027	Japan	29,902	38.02	Oronasopharynx	10 February 2020
3	LC529905.1	TKYE6182_2020	Japan	29,903	37.97	NA^*a*^	January 2020
4	LR757995.1	Wuhan seafood market pneumonia virus	China (Wuhan)	29,872	38	NA	5 January 2020
5	MT163720.1	WA8-UW5/human/2020/USA	United States	29,732	37.97	NA	1 March 2020
6	LR757998.1	Wuhan seafood market pneumonia virus	China (Wuhan)	29,866	37.99	NA	26 December 2019
7	MN908947.3	Wuhan-Hu-1	China	29,903	37.97	NA	December 2019
8	MN938384.1	2019-nCoV_HKU-SZ-002a_2020	China (Shenzhen)	29,838	38.02	Oronasopharynx	10 January 2020
9	MN975262.1	2019-nCoV_HKU-SZ-005b_2020	China	29,891	37.98	Oronasopharynx	11 January 2020
10	MN985325.1	2019-nCoV/USA-WA1/2020	United States	29,882	38	Oronasopharynx	19 January 2020
11	MN988668.1	2019-nCoV WHU01	China	29,881	38	NA	2 January 2020
12	MN988669.1	2019-nCoV WHU02	China	29,881	38	NA	2 January 2020
13	MN988713.1	2019-nCoV/USA-IL1/2020	United States	29,882	37.99	Lung, oronasopharynx	21 January 2020
14	MN994467.1	2019-nCoV/USA-CA1/2020	United States	29,882	38	Oronasopharynx	23 December 2019
15	MN994468.1	2019-nCoV/USA-CA2/2020	United States	29,883	37.99	Oronasopharynx	22 January 2020
16	MN996527.1	WIV02	China	29,825	38.02	Lung	30 December 2019
17	MN996528.1	WIV04	China	29,891	37.99	Lung	30 December 2019
18	MN996529.1	WIV05	China	29,852	38.02	Lung	30 December 2019
19	MN996530.1	WIV06	China	29,854	38.03	Lung	30 December 2019
20	MN996531.1	WIV07	China	29,857	38.02	Lung	30 December 2019
21	MN997409.1	2019-nCoV/USA-AZ1/2020	United States	29,882	37.99	Feces	22 January 2020
22	MT007544.1	Australia/VIC01/2020	Australia	29,893	37.97	NA	25 January 2020
23	MT012098.1	SARS-CoV-2/29/human/2020/IND	Kerala, India	29,854	38.02	Oronasopharynx	27 January 2020
24	MT019529.1	BetaCoV/Wuhan/IPBCAMS-WH-01/2019	China	29,899	37.98	Lung	23 December 2019
25	MT019530.1	BetaCoV/Wuhan/IPBCAMS-WH-02/2019	China	29,889	38	Lung	30 December 2019
26	MT019531.1	BetaCoV/Wuhan/IPBCAMS-WH-03/2019	China	29,899	37.98	Lung	30 December 2019
27	MT019532.1	BetaCoV/Wuhan/IPBCAMS-WH-04/2019	China	29,890	37.99	Lung	30 December 2019
28	MT019533.1	BetaCoV/Wuhan/IPBCAMS-WH-05/2020	China	29,883	37.99	Lung	1 January 2020
29	MT020880.1	2019-nCoV/USA-WA1-A12/2020	United States	29,882	38	Oronasopharynx	25 January 2020
30	MT020881.1	2019-nCoV/USA-WA1-F6/2020	United States	29,882	38	Oronasopharynx	25 January 2020
31	MT027062.1	2019-nCoV/USA-CA3/2020	United States	29,882	38	Oronasopharynx	29 January 2020
32	MT027063.1	2019-nCoV/USA-CA4/2020	United States	29,882	38	Oronasopharynx	29 January 2020
33	MT027064.1	2019-nCoV/USA-CA5/2020	United States	29,882	37.99	Oronasopharynx	29 January 2020
34	MT039873.1	HZ-1	China	29,833	38.02	Lung, Oronasopharynx	20 January 2020
35	MT039887.1	2019-nCoV/USA-WI1/2020	United States	29,879	38	Oronasopharynx	31 January 2020
36	MT039888.1	2019-nCoV/USA-MA1/2020	United States	29,882	37.99	Oronasopharynx	29 January 2020
37	MT039890.1	SNU01	South Korea	29,903	37.96	NA	January 2020
38	MT044257.1	2019-nCoV/USA-IL2/2020	United States	29,882	38	Lung, Oronasopharynx	28 January 2020
39	MT044258.1	2019-nCoV/USA-CA6/2020	United States	29,858	38	Oronasopharynx	27 January 2020
40	MT049951.1	SARS-CoV-2/Yunnan-01/human/ 2020/CHN	China	29,903	37.97	Lung, Oronasopharynx	17 January 2020
41	MT050493.1	SARS-CoV-2/166/human/2020/IND	Kerala, India	29,851	38.01	Oronasopharynx	31 January 2020
42	MT066156.1	SARS-CoV-2/NM	Italy	29,867	38.01	Lung, Oronasopharynx	30 January 2020
43	MT066175.1	SARS-CoV-2/NTU01/2020/TWN	Taiwan	29,870	38.01	NA	31 January 2020
44	MT066176.1	SARS-CoV-2/NTU02/2020/TWN	Taiwan	29,870	38.01	NA	5 February 2020
45	MT072688.1	SARS0CoV-2/61-TW/human/2020/ NPL	Nepal	29,811	38.02	Oronasopharynx	13 February 2020
46	MT093571.1	SARS-CoV-2/01/human/2020/SWE	Sweden	29,886	38	NA	7 February 2020
47	MT093631.2	SARS-CoV-2/WH-09/human/2020/CHN	China	29,860	38.02	Oronasopharynx	8 January 2020
48	MT106052.1	2019-nCoV/USA-CA7/2020	United States	29,882	37.99	Oronasopharynx	6 February 2020
49	MT106053.1	2019-nCoV/USA-CA8/2020	United States (CA)	29,882	38	Oronasopharynx	10 February 2020
50	MT106054.1	2019-nCoV/USA-TX1/2020	United States (TX)	29,882	38	Lung, Oronasopharynx	11 February 2020
51	MT118835.1	2019-nCoV/USA-CA9/2020	United States (CA)	29,882	38	Lung	23 February 2020
52	MT121215.1	SARS-CoV-2/SH01/human/2020/CHN	China	29,945	37.91	Oronasopharynx	2 February 2020
53	MT123290.1	SARS-CoV-2/IQTC01/human/2020/CHN	China	29,891	38	Oronasopharynx	5 February 2020
54	MT123291.2	SARS-CoV-2/IQTC02/human/2020/CHN	China	29,882	37.99	Lung	29 January 2020
55	MT123292.2	SARS-CoV-2/QT	China	29,923	38.02	Lung, Oronasopharynx	27 January 2020
56	MT123293.2	SARS-CoV-2/IQTC03/human/2020/CHN	China	29,871	38	Feces	29 January 2020
57	MT126808.1	SARS-CoV-2/SP02/human/2020/BRA	Brazil	29,876	38	Oronasopharynx	28 February 2020
58	MT135041.1	SARS-CoV-2/105/human/2020/CHN	China:Beijing	29,903	37.97	NA	26 January 2020
59	MT135042.1	SARS-CoV-2/231/human/2020/CHN	China:Beijing	29,903	37.97	NA	28 January 2020
60	MT135043.1	SARS-CoV-2/233/human/2020/CHN	China:Beijing	29,903	37.97	NA	28 January 2020
61	MT135044.1	SARS-CoV-2/235/human/2020/CHN	China:Beijing	29,903	37.97	NA	28 January 2020
62	MT152824.1	SARS-CoV-2/WA2/human/2020/USA	United States (WA)	29,878	38	Mid-nasal swab	24 February 2020
63	MT159705.1	2019-nCoV/USA-CruiseA-7/2020	United States	29,882	37.99	Oronasopharynx	17 February 2020
64	MT159706.1	2019-nCoV/USA-CruiseA-8/2020	United States	29,882	38	Oronasopharynx	17 February 2020
65	MT159707.1	2019-nCoV/USA-CruiseA-10/2020	United States	29,882	38	Oronasopharynx	17 February 2020
66	MT159708.1	2019-nCoV/USA-CruiseA-11/2020	United States	29,882	38	Oronasopharynx	17 February 2020
67	MT159709.1	2019-nCoV/USA-CruiseA-12/2020	United States	29,882	38	Oronasopharynx	20 February 2020
68	MT159710.1	2019-nCoV/USA-CruiseA-9/2020	United States	29,882	38	Oronasopharynx	17 February 2020
69	MT159711.1	2019-nCoV/USA-CruiseA-13/2020	United States	29,882	38	Oronasopharynx	20 February 2020
70	MT159712.1	2019-nCoV/USA-CruiseA-14/2020	United States	29,882	37.99	Oronasopharynx	25 February 2020
71	MT159713.1	2019-nCoV/USA-CruiseA-15/2020	United States	29,882	38	Oronasopharynx	18 February 2020
72	MT159714.1	2019-nCoV/USA-CruiseA-16/2020	United States	29,882	38	Oronasopharynx	18 February 2020
73	MT159715.1	2019-nCoV/USA-CruiseA-17/2020	United States	29,882	38	Oronasopharynx	24 February 2020
74	MT159716.1	2019-nCoV/USA-CruiseA-18/2020	United States	29,867	38	Oronasopharynx	24 February 2020
75	MT159717.1	2019-nCoV/USA-CruiseA-1/2020	United States	29,882	37.99	Oronasopharynx	17 February 2020
76	MT159718.1	2019-nCoV/USA-CruiseA-2/2020	United States	29,882	37.99	Oronasopharynx	18 February 2020
77	MT159719.1	2019-nCoV/USA-CruiseA-3/2020	United States	29,882	38	Oronasopharynx	18 February 2020
78	MT159720.1	2019-nCoV/USA-CruiseA-4/2020	United States	29,882	37.99	Oronasopharynx	21 February 2020
79	MT159721.1	2019-nCoV/USA-CruiseA-5/2020	United States	29,882	38	Oronasopharynx	21 February 2020
80	MT159722.1	2019-nCoV/USA-CruiseA-6/2020	United States	29,882	37.99	Oronasopharynx	21 February 2020
81	MT163716.1	SARS-CoV-2/WA3-UW1/human/ 2020/USA	United States (WA)	29,903	37.95	NA	27 February 2020
82	MT163717.1	SARS-CoV-2/WA4-UW2/human/ 2020/USA	United States (WA)	29,897	37.97	NA	28 February 2020
83	MT163718.1	SARS-CoV-2/WA6-UW3/human/ 2020/USA	United States (WA)	29,903	37.97	NA	29 February 2020
84	MT163719.1	SARS-CoV-2/WA7-UW4/human/ 2020/USA	United States (WA)	29,903	37.97	NA	1 March 2020
85	LR757996.1	Wuhan seafood market pneumonia virus	China (Wuhan)	29,732	37.96	NA	1 January 2020
86	MT184907.1	2019-nCoV/USA-CruiseA-19/2020	United States	29,882	38	Oronasopharynx	18 February 2020
87	MT184908.1	2019-nCoV/USA-CruiseA-21/2020	United States	29,880	38	Oronasopharynx	17 February 2020
88	MT184909.1	2019-nCoV/USA-CruiseA-22/2020	United States	29,882	38	Oronasopharynx	21 February 2020
89	MT184910.1	2019-nCoV/USA-CruiseA-23/2020	United States	29,882	37.99	Oronasopharynx	18 February 2020
90	MT184911.1	2019-nCoV/USA-CruiseA-24/2020	United States	29,882	37.97	Oronasopharynx	17 February 2020
91	MT184912.1	2019-nCoV/USA-CruiseA-25/2020	United States	29,882	38	Oronasopharynx	17 February 2020
92	MT184913.1	2019-nCoV/USA-CruiseA-26/2020	United States	29,882	37.99	Oronasopharynx	24 February 2020
93	MT188339.1	USA/MN3-MDH3/2020	United States (MN)	29,783	38.01	Oronasopharynx	7 March 2020
94	MT188340.1	USA/MN2-MDH2/2020	United States (MN)	29,845	37.98	Oronasopharynx	9 March 2020
95	MT188341.1	USA/MN1-MDH1/2020	United States (MN)	29,835	37.99	Oronasopharynx	5 March 2020

### Phylogenomic analysis: defining evolutionary relatedness.

**FIG 1:**
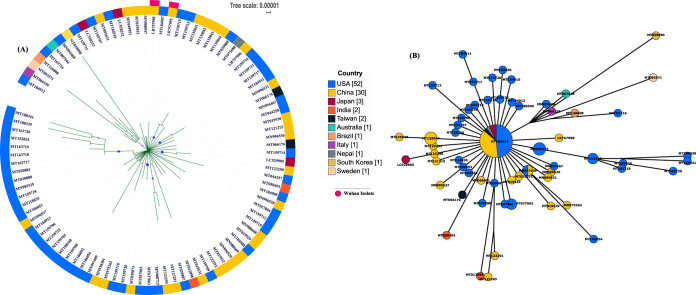
(A) Core genome-based phylogenetic analysis of SARS-CoV-2 isolates using the maximum likelihood method based on the Tamura-Nei model. The analysis involved 95 SARS-CoV-2 sequences with a total of 28,451 nucleotide positions. Bootstrap values of more than 70% are shown on branches as blue dots with sizes corresponding to the bootstrap values. The colored circle represents the country of origin of each isolate. The two isolates from Wuhan are marked separately on the outer side of the ring. (B) The minimum spanning tree generated using maximum likelihood method and Tamura-Nei model showing the genetic relationships of SARS-CoV-2 isolates with their geographical distribution.

**FIG 2:**
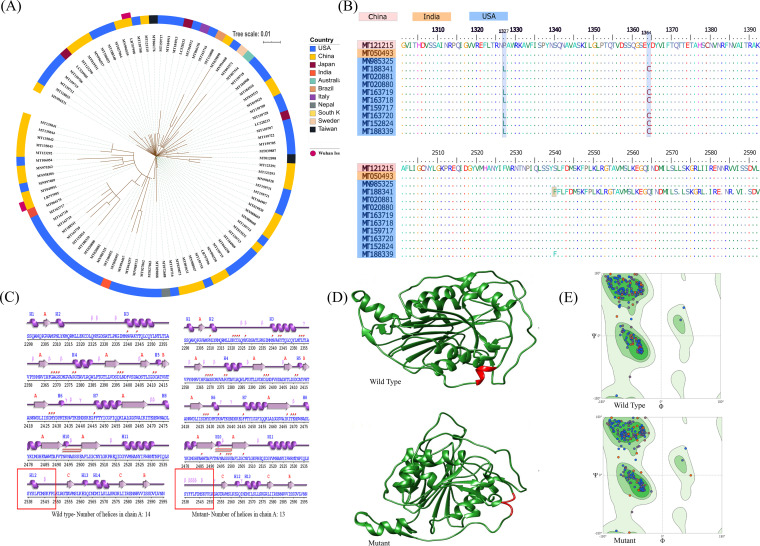
(A) SNP-based phylogeny of SARS-CoV-2 isolates. Highly similar genomes of coronaviruses were taken as the input by Parsnp. Whole-genome alignments were made using libMUSCLE aligner and the annotated genome of MT121215 strain as the reference. Parsnp identifies the maximal unique matches (MUMs) among the query genomes provided in a single directory. As only the genomes corresponding to a specified MUM index (MUMI) distance threshold are recruited, option -c was used to force inclusion of all the strains. The output phylogeny based on single nucleotide polymorphisms was obtained following variant calling on core-genome alignment. (B) Multiple-sequence alignment of ORF1b protein showing amino acid substitutions at three positions: P1327L, Y1364C, and S2540F. The isolate USA/MN1-MDH1/2020 (MT188341) showed an amino acid addition leading to a change in an amino acid frame from position 2540 onward. (C and D) 2D and 3D structures for nsp16 in the wild-type strain (MT121215) and the mutant strain (MT188341) predicted using PDBsum and SWISS-MODEL. (E) Ramachandran plot of the predicted wild-type and mutant proteins, where the green region represents a most-favored region whereas the light green area denotes an allowed region. The white zone represents a generously allowed region.

Our analysis revealed that strains of human infecting SARS-CoV-2 are novel and highly similar (>99.9%). A recent study established the closest neighbor of SARS-CoV-2 to be SARSr-CoV-RaTG13, a bat coronavirus (25). As COVID-19 transitioned from epidemic to pandemic due to the extremely contagious nature of the SARS-CoV-2, it was interesting to delineate the relationship between strains and their geographical locations. In this study, we employed two methods to delineate the phylogenomic relatedness of the isolates: analyses of the core genome (Fig. 1) and single nucleotide polymorphisms (SNPs) (Fig. 2A). The phylogenies obtained were annotated with the country of isolation of each strain (Fig. 1). Using GrapeTree, we identified strains which were diverging from the common core population (Fig. 1B). For example, two isolates from the United States (GenBank accession no. MN994468 and MT163716) and one each from Australia (MT007544), Italy (MT066156), Sweden (MT093571), South Korea (MT039890), and Brazil (MT126808) were found to have diverged from a recent common ancestor (Fig. 1B). Further, the phylogenetic clustering was found majorly concordant by both the core-genome-based (Fig. 1A) and the SNP-based (Fig. 2A) methods. The strains formed a monophyletic clade, in which MT039890 (Sweden) and MT093571 (South Korea) were the most diverged. Focusing on the edge connection between the neighboring countries from which the transmission is more likely to occur, we noted that a strain from Taiwan (MT066176) clustered closely with another strain from China (MT121215). With the exception of those two strains, we did not find any connection between strains of neighboring countries. Thus, most strains belonging to the same country clustered distantly from each other and showed relatedness to strains isolated from distant geographical locations (Fig. 1A; see also Fig. 2A). For instance, a SARS-CoV-2 strain isolated from Nepal (MT072688) clustered with a strain from the United States (MT039888). Also, strains from Wuhan (GenBank accession no. LR757998 and LR757995), where the virus was originated, showed highest identity with the United States strains as well as the Chinese strains; strains MT012098 and MT050493 from India (26) clustered closely with China and U.S. strains, respectively (Fig. 1A; see also Fig. 2A). Similarly, an Australian strain (MT007544) showed close clustering with a U.S. strain (Fig. 1A; see also Fig. 2A) and one strain from Taiwan (MT066175) clustered with highly similar Chinese isolates (Fig. 2A). Isolates from Italy (MT012098) and Brazil (MT126808) clustered with different U.S. strains (Fig. 1A; see also Fig. 2A). Notably, isolates from same country or geographical location formed a mosaic pattern of phylogenetic placements of isolates from those countries. For viral transmission, contact between the individuals is also an important factor, due to which the spread of identical strains across the border of neighboring countries is supposedly more likely. But we obtained a pattern where the Indian strains showed highest similarity with the United States and China strains, the Australian strains with the United States strains, and the Italian and Brazilian strains with strains isolated from the United States, among others. This depicts the viral spread across different communities. However, as SARS-CoV-2 genomes were available mostly from the United States and China, sampling biases are evident in the analyzed data set available on NCBI. Thus, it is plausible for strains from other countries to show the highest similarity to strains from these two countries. In the near future, as more and more genome sequences become available from different geographical locations, more-accurate patterns representing their relatedness across the globe will become available.

Recent studies have also focused on the roles of synanthropic animals in transmission of SARS-COV-2 where epitopes of spike and nucleocapsid proteins of taxonomically related coronaviruses of animal species living in close vicinity to humans were compared with those of SARS-COV-2 (27, 28). Here, we also compared the spike and nucleocapsid proteins of other synanthropic animals with those of 95 SARS-CoV-2 strains (see Fig. S1A and B in the supplemental material). Phylogenetic clustering revealed that all the SARS-CoV-2 strains formed a clear-cut separate cluster (Fig. S1A and 1B). The results obtained suggested that the homology of spike and nucleocapsid proteins of SARS-CoV-2 with those of other animal coronaviruses was accountable, but not much higher. This supports the hypothesis that the synanthropic animals may not act as a reservoir for the infection but may rather provide a stimulus for an immune response which may help the organism to fight against SARS-CoV-2 (27, 28). On the other hand, the recurring exposure might lead to eliciting overresponsiveness by the immune system and thus might worsen the symptoms.

10.1128/mSystems.00505-20.1 FIG S1 Phylogeny construction of (A) nucleocapsid and (B) spike proteins of 95 SARS-CoV2 strains isolated from synanthropic animals. The accession numbers of the proteins are given in parentheses. The sequences were aligned using the MUSCLE (76) aligner, and phylogeny was constructed at MEGAX using the neighbor joining method (73) and visualized in interactive Tree of Life (iTOL) (74). Download FIG S1, PDF file, 0.04 MB. Copyright © 2020 Kumar et al. 2020 Kumar et al. This content is distributed under the terms of the Creative Commons Attribution 4.0 International license.

### SNPs in the SARS-CoV-2 genomes.

SNPs in all predicted ORFs in each genome were analyzed using SARS-CoV-2/SH01/human/2020/CHN as a reference. SNPs were assayed using maximum unique matches between the genomes of coronavirus. We observed that the strains isolated from the United States (MT188341, MN985325, MT020881, MT020880, MT163719, MT163718, MT163717, MT152824, MT163720, and MT188339) are the most evolved and that they carry set of unique point mutations (Table 2) in nsp13, nsp14, nsp15, nsp16 (present in the orf1b polyprotein region), and S-protein (Table 2; see also Table S1 in the supplemental material). Most of these mutated proteins are nonstructural proteins (NSP) functionally involved in forming viral replication-transcription complexes (RTC) (29). For instance, nsp13 belongs to helicase superfamily 1 and is putatively involved in viral RNA replication through RNA-DNA duplex unwinding (30) whereas nsp14 and nsp15 represent an exoribonuclease and an endoribonuclease, respectively (31, 32). nsp16 functions as mRNA cap-1 methyltransferase (33). All these proteins contain SNPs at several positions (Table 2), which indicates that the viral machinery for its RNA replication and processing had evolved to the utmost in strains from the United States compared to those from the other countries. Further, we analyzed the SNPs at the protein level; interestingly, there were amino acid substitutions at P1327L, Y1364C, and S2540F in the ORF1b protein in U.S. isolates.

**TABLE 2:** Major mutations present in different isolates of SARS-CoV-2 at different locations

Strain(s) with major mutation(s)	Protein	Position in reference genome	Variant nucleotide different from reference	Nucleotide in reference genome
MT188341; MN985325; MT020881; MT020880; MT163719; MT163718; MT163717; MT152824; MT163720; MT188339	NSP14	18060	T	C
MT188341; MT163719; MT163718; MT163717; MT152824; MT163720; MT188339	NSP13	17747	T	C
MT188341; MT163719; MT163718; MT163717; MT152824; MT163720; MT188339	NSP13	17858	G	A
MT188341	NSP13	16467	G	A
Several strains under study	NSP3	6026	C	T
MT039888	NSP3	3518	T	G
MT039888	NSP3	17423	G	A
MT163719	NSP15	20281	G	T
MT188339	NSP16	21147	C	T
MT188341	S-protein	23185	T	C
MT163720	S-protein	23525	T	C
MT188339	S-protein	22432	T	C
MT159716	S-protein	22033	A	C
MT050493 (Indian)	S-protein	24351	T	C

a NA, information not available.

10.1128/mSystems.00505-20.3 TABLE S1 The amino acid mutations in S-protein of SARS-CoV-2 isolates. Download Table S1, DOCX file, 0.01 MB. Copyright © 2020 Kumar et al. 2020 Kumar et al. This content is distributed under the terms of the Creative Commons Attribution 4.0 International license.

One isolate, namely, USA0/MN1-MDH1/2020 (MT188341), carried an amino acid addition at position 2540 leading to a shift in the amino acid frame from there onward (Fig. 2B). The insertion of phenylalanine in the sequence might result in an increased size of the mutant residue compared to the wild-type residue, which might affect the functioning of nsp16 (2′-O-MTase). To further analyze the structural heterogeneity resulting from the insertion of phenylalanine, we predicted the two-dimensional (2D) and 3D structures of the wild type (MT121215) and the mutant (MT188341) based on homology modeling with identity levels of 100% for the reference sequence (template: 6w61.1.A) and 99.66% for the mutant sequence. Interestingly, the protein structures were found to be different (Fig. 2C and D). We predicted the motifs in these strains and observed that one motif from mutant strain at positions 243 to 246 was missing compared to the wild type (Fig. 2C). Thus, the results suggested the presence of 14 helical regions in the wild-type strain, whereas only 13 helices were present in the mutant type (Fig. 2C). The region of mutation/insertion that lacked the helical region was found to harbor beta sheets (Fig. 2C). Further, the 3D structure analysis results showing sequence identity above 99% revealed that the model was constructed with a high confidence value (Fig. 2D). Thereafter, we validated the structures with the help of Ramachandran plot analysis. In case of the wild type, the analysis predicted the presence of 97.97% residues in the favored region, in contrast to the presence of 95.98% residues in the favored region in the mutant (Fig. 2E). Thus, we concluded that the wild-type structure is more stable than the mutant structure. Further, the results of the protein stability analysis showed that this mutation could decrease the stability of this protein with possible effects on size and hydrophobicity. This mutant residue was found to be more hydrophobic than the wild-type residue, which would impact hydrogen bond formation. The residue in the wild type is buried in the core of a domain. The differences between the wild-type and mutant residues might disturb the core structure of this domain. Thus, in our opinion the mutation may lead to a conformational change in the protein structure and affect the functioning of nsp16.

Additionally, we found two dominant mutations in the nsp13 protein (P1327L and Y1364C mutations in orf1b) which codes for helicase enzyme in six American isolates. Analysis performed using the HOPE server revealed that the mutation at position 1364, where tyrosine was replaced with cysteine, decreased the affinity of helicase for the RNA template, while the replacement of proline with leucine increased the affinity of helicase for the RNA template. Thus, taking the results together, mutations reduced the global values of the mutant protein (0.77 ± 0.05) compared to the wild type (0.79 ± 0.05); however, there was no change seen in the topology of the protein structure. As these rapidly evolving proteins are involved in viral replication, the mutations need to be considered in developing a vaccine.

### Host pathogenic interactions and functional analysis.

**FIG 3:**
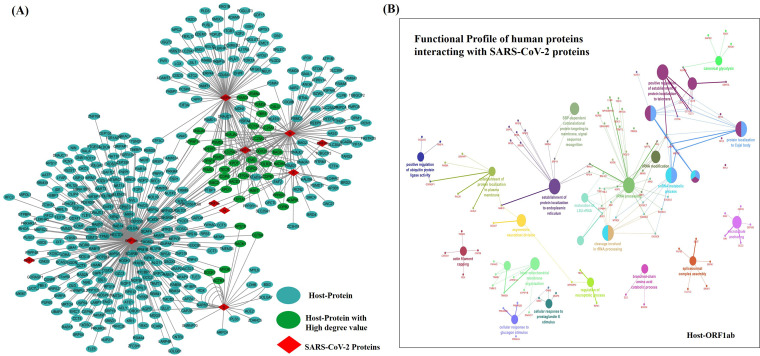
SARS-CoV-2–host interactome and its functional annotation. (A) SARS-CoV-2-host interaction map predicted using the IntAct database, showing human proteins interacting with 10 viral proteins. (B) Gene ontology (GO) analysis was performed for host proteins interacting with ORF1ab using the ClueGo Cytoscape app against database KEGG, the Gene Ontology—biological function database, and Reactome pathways. ClueGo parameters were set as follows: Go Term Fusion selected; *P* values of ≤0.05; GO tree interval, all levels; kappa score of 0.42.

We analyzed the host-pathogen interactions between SARS-CoV-2 and human proteins. Analyses performed with the IntAct database (34–36) revealed that of 10 viral proteins, 8 showed significant interactions with human proteins. Among those eight proteins, six (the ORF1ab, NSP8, M, ORF7a, S, and E proteins) showed significant functional interactions (Bonferroni correction; *P* < 0.05) with 248, 89, 86, 48, 19, and 14 host proteins, respectively, resulting into a total of 396 nodes and 521 edges (Fig. 3; see also Table S2). The Gene Ontology (GO)-based functional annotations of complex network revealed that the major host pathways that were manipulated by the viral proteins included those involved in regulation of metabolic processes, protein localizations, nucleus export, rRNA processing, stress responses, etc. As mentioned above, Orf1ab encodes a total of 16 nonstructural proteins (nsp1 to nsp16) which constitute a replicase/transcriptase complex (RTC) (37). These nonstructural proteins were found to interact with 248 host proteins involved in multiple intracellular pathways (Fig. 3; see also Table S2).

10.1128/mSystems.00505-20.4 TABLE S2 List of host (human) proteins showing significant interaction with viral proteins. Download Table S2, XLSX file, 0.02 MB. Copyright © 2020 Kumar et al. 2020 Kumar et al. This content is distributed under the terms of the Creative Commons Attribution 4.0 International license.

The interactome data revealed that the interferon signaling pathway is targeted by orf1ab during infection. The interaction is mediated by Tank binding kinase-1 (TBK1) binding protein, which constitutively binds with TBK1 and inhibitor of NF-κB kinase subunit epsilon (IKBKE), which are crucial for mediating the antiviral immune responses (38, 39). Furthermore, the interaction of orf1ab with Transducin-like enhancer protein 1 (TLE1), which is a transcriptional corepressor of NF-κB (40), confirms the involvement of NF-κB signaling during SARS-CoV-2 infection. It is now well established that COVID-19 pathogenesis is driven by profound cytokines responses such as those of interleukin-6 (IL-6), IL-8, tumor necrosis factor (TNF), IL-1β, granulocyte colony-stimulating factor (G-CSF), granulocyte-macrophage colony-stimulating factor (GM-CSF), etc. (41). Reports suggest that a phase II clinical trial is focusing on neutralizing IL-8 in order to improve the health condition of COVID-19 patients (https://clinicaltrials.gov/ct2/show/NCT04347226). We also found that SARS-COV-2 protein (orf1ab) interacts with NF-κB-repressing factor (NKRF), which is a potential regulator of IL-8; thus, targeting this interaction may subsequently improve the health condition of COVID-19 patients.

Further, heterogeneous RNA molecules such as snoRNA/small Cajal body-specific RNA (scaRNA) and snRNA, which are located in the Cajal body (in regions within the nucleus that are enriched in RNAs and proteins) have been reported to facilitate the activity of 2′-O-ribose-methylated nucleotides and pseudouridines in the RNA polymerase II-transcribed U1, U2, U4 and U5 spliceosomes (42). Through our interactome studies, we found that, remarkably, the multiple spliceosome components [SLU7, poly(U)-binding-splicing factor 60 (PUF60), SRSF protein kinase 1 (SRPK1), SRSF5, LUC7L2, U2AF1L5, SNRNP70, HNRNPUL2, etc.] of the host interact with viral proteins (Fig. 3; see also Table S2). Although previous studies showed that the role of interactions between spliceosome components and host proteins was substantial in SARS-CoV infections and other coronavirus infections (43, 44), with this study, we confirmed their role in novel coronavirus (SARS-CoV-2) infection, too. We found that orf1ab interacts with SRSF protein kinase 1 (SRPK1), which plays a central role in splicing and is known to phosphorylate serine/arginine-rich splicing factor 1 (SRSF1) (45). SRSF1 regulates the accuracy of splicing and also that of alternative splicing. PUF60, another component of the spliceosome, was found to interact with orf1ab, which plays a vital role in pre-mRNA splicing and 3′ end processing (46). It promotes the splicing of introns in a cooperative manner with another splicing factor, U2AF2 (46). The U4/U6-U5 tri-snRNP complex is involved in spliceosome assembly, and one component, U4/U6 small nuclear ribonucleoprotein Prp3 (PRPF3) (47), was found to interact with orf1ab. The results of our analysis emphasized that SARS-CoV-2 manipulates spliceosome machinery during infection; hence, targeting splicing might affect viral replication. Recently, Bojkova et al. also showed that addition of spliceosome inhibitor Pladeinolide-B into SARS-CoV-2-infected human Caco-2 cells significantly inhibited viral replication (48). Thus, targeting the splicing could be another prospective drug discovery.

Further, there are reports which suggest that targeting notch signaling could be a way to prevent SARS-CoV-2 infection, as notch-mediated downregulation of furin (a host protease) levels was found to interfere with entrance of the virus into the host cell (49). Interestingly, we showed that nsp8 interacts with POGLUT2, POGLUT3 and POFUT1, which regulate the transport of notch1 and notch3 to the plasma membrane and fucosylation of notch1 protein, thereby modulating the notch signaling (50, 51).

Membrane (M) and envelope (E) proteins are structural proteins which are crucial for viral assembly and pathogenesis. Our interactome studies showed that M and E proteins of SARS-CoV-2 interact with multiple host proteins. Membrane protein and nsp8 have been found to interact with protein transport protein Sec16A (Fig. S2; see also Table S2), which mediates endoplasmic reticulum (ER) membrane insertion of SARS-CoV-2 proteins, critical for cotranslational entry into secretory pathways (52). Further, the data also showed that M and nsp8 proteins interact with host protein responsible in cristae formation, which suggests the possibility of mitochondrial fusion-mediated downregulation of host cell interferon gamma responses similar to that seen with SARS-COV (53). A similar form of mitochondrial dysfunction in SARS-CoV-2 infected cells was also reported previously (54).

10.1128/mSystems.00505-20.2 FIG S2 Functional analysis of SARS-CoV-2–host interactome. Gene ontology (GO) analysis was performed for host proteins interacting with M, NSP8, E, S, and ORF7a by the use of the ClueGo Cytoscape app against database KEGG, Gene Ontology (biological function database), and Reactome pathways. Download FIG S2, EPS file, 0.7 MB. Copyright © 2020 Kumar et al. 2020 Kumar et al. This content is distributed under the terms of the Creative Commons Attribution 4.0 International license.

Furthermore, we showed that the E-protein interacts with bromodomain proteins (BRD4), which is in agreement with a previous study in which the researchers showed that SARS-CoV-2 envelop interacted with bromodomain proteins BRD2 and BRD4 to regulate gene transcription (52). Unfortunately, the later stages of COVID-19 infection result in development of a hypoxic condition which leads to progression of ARDS (acute respiratory distress syndrome) and toxic encephalopathy (55). Our interactome study revealed that ORF7a potentially regulates such hypoxic conditions by interacting with host proteins. Studies have shown that kidney involvement is frequent in COVID-19 patients and even acute kidney injury is common in critically ill patients (56). Our interactome analysis showed an interaction of ORF7a and nsp8 with NPEPPS, a puromycin-sensitive amino peptidase, which is commonly used as a biomarker against damaged kidneys (57). Our results also indicated the interaction of ORF7a and nsp8 with multiple proteasome-related proteins such as PSMD6, PSMD7, PSMD2, and PSMD13, which is in agreement with a previous study in which the authors revealed the interactions by overexpressing the SARS-CoV-2 gene in HEK293 cells (58). Taking the results together, these proteins regulate multiple cellular pathways, such as interleukin pathways, which may be a reason for the high plasma concentrations of cytokines such as IL-2, IL-7, IL-10, and IL-6 in critically ill COVID-19 patients (59), and indeed, the cytokine storm is a major cause of inflammatory cascades during COVID-19 infection (60).

It is well known that spike (S) glycoproteins facilitate the entry of SARS-CoV-2 into host cells by binding with cellular receptor angiotensin-converting enzyme 2 (ACE2) (2, 61, 62). This protein has been the most highly studied protein so far, and our interactome results are in consensus with the previous reports. Moreover, the studies on HEK293T cells have shown that the S-proteins of SARS-CoV-2 were entirely processed at the S1 and S2 sites during biosynthesis in the Golgi compartment (2). We also found strong interactions among S-protein, ZDHHC5, and GOLGA7 (Fig. S2), where the latter two formed a palmitoyltransferase complex involved in palmitoylation process and in transport from the Golgi compartment to the cell surface. S-proteins also interact with LDHB (lactate dehydrogenase B), a hallmark of inflammation during COVID-19 infection. Recent studies of critically ill COVID-19 patients showed high levels of lactose dehydrogenase (63, 64). It is presumed that increased LDH levels lead to the complexity of disease manifestations accompanied by respiratory failure due to prolonged severe inflammatory responses. Being the linchpin in the process, it is considered to represent a possible biomarker for diagnostic testing to identify persons infected with SARS-CoV-2 (63–65).

Taking the results together, this study integrated the host-pathogen protein interaction network using data sets generated from different studies (35, 36, 52) and showed that SARS-CoV-2 viral proteins discretely manipulate the host system for their own survival and pathogenicity.

### Direction of selection of SARS-CoV-2 genes.

**FIG 4:**
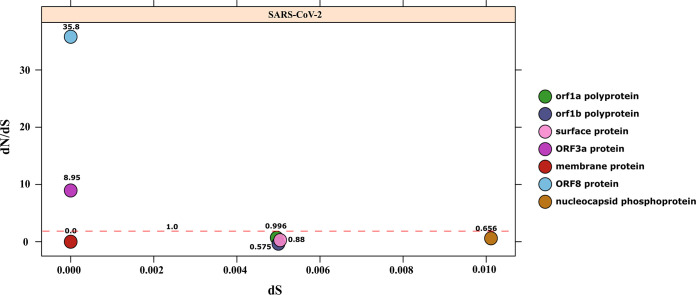
Estimation of purifying natural selection pressure in nine coding sequences of SARS-CoV-2. *dN*/*dS* values are plotted as a function of *dS*.

Our analysis revealed that ORF8 (121 amino acids [aa]) (*dN*/*dS* = 35.8), along with ORF3a (275 bp), (*dN*/*dS* = 8.95) showed the highest *dN*/*dS* values among the nine ORFs and that ORF8 and ORF3a thus have much greater numbers of nonsynonymous substitutions than the synonymous substitution (Fig. 4). *dN*/*dS* values that are much higher than 1 are indicative of a strongly divergent lineage (66). Thus, both of these proteins are evolving under conditions of high selection pressure and represent ORFs that are highly divergent across strains. Two other proteins, ORF1ab polyprotein (*dN*/*dS* = 0.996 and 0.575) and S-protein (*dN*/*dS* = 0.88) might confer a selective advantage with respect to host challenges and survival. The *dN*/*dS* rate values of nearly 1 and greater than 1 suggest that the strains are coping well with the challenges that they encounter, i.e., with immune responses and the inhibitory environment of host cells (67). The other gene clusters, namely, those corresponding to M-protein and orf1a polyprotein, did not possess at least three unique sequences, which is a condition that is necessary for the analysis; hence, they should be similar across the strains. The two ORF1ab polyprotein genes that code for protein translation and posttranslation modification were found to have evolved to perform active translation and to facilitate the multiplication and growth of virus inside the host. Similarly, the S-protein which helps in the entry of virus into the host cells by facilitating penetration of the cell membrane was found to be involved in the acceleration toward positive selection, confirming the ability of the enzyme to initiate the infection. Another positively diversifying N protein gene codes for nucleocapsid formation, which protects the genetic material of virus from host immune responses such as cellular protease activity. Overall, the data indicate that the growth-related and multiplication-related genes are evolving at a high rate. The results revealing other proteins with *dN*/*dS* values equal to zero suggest a conserved repertoire of genes.

### Conclusions.

As COVID-19 continues to impact virtually all human lives worldwide due to its extremely contagious nature, it has spiked the interest of scientific community all over the world in better understanding of the pathogenesis of the novel SARS-CoV-2 virus. In this study, analyses were performed on the genomes of the novel SARS-CoV-2 isolates recently reported from different countries to understand the viral pathogenesis. We observed no direct pattern of transmission of the novel SARS-CoV-2 in the neighboring countries through our analyses of the phylogenomic relatedness of geographical isolates. Isolates collected from same locations, for instance, isolates from the United States and isolates from China, were phylogenetically distant. Thus, there appears to be a mosaic pattern of transmission indicating that it represents the result of travel of infected humans among different countries. As COVID-19 transited from epidemic to pandemic within a short time, such a result does not look surprising on the basis of the genome structures of the viral isolates. The genomes of six isolates, specifically, from the United States, were found to harbor unique amino acid SNPs and showed amino acid substitutions in ORF1b protein and S-protein, while one of them also harbored an amino acid addition. The ORF1ab polyprotein and S-protein were also found to have *dN*/*dS* values approaching 1 and thus might confer a selective advantage enabling the virus to evade host response mechanisms. Thus, these proteins are rapidly evolving and are involved in viral replication. Therefore, these mutations cannot be neglected while developing a vaccine. Further, the SARS-CoV-2–human interactome revealed that its pathogenicity is mediated by a surge in proinflammatory cytokine levels. It is predicted that the major mechanism of immune pathogenicity in SARS-CoV-2 includes alteration of the host cell environment by disintegration of signal transduction pathways and immunity evasion by several protection mechanisms. Our results provide insights into COVID-19 genomes and interactomes which may be useful to understand the impact of virus on human health.

## MATERIALS AND METHODS

### Selection of genomes and annotation.

Sequences of different strains were downloaded from NCBI database https://www.ncbi.nlm.nih.gov/genbank/sars-cov-2-seqs/ (Table 1). A total of 97 genomes were downloaded on 19 March 2020 from the NCBI database, and, based on quality assessment, two genomes with multiple Ns were removed from the study. Further, the genomes were annotated using Prokka (68). A manually annotated reference database was generated using the GenBank file of severe acute respiratory syndrome coronavirus 2 isolate SARS-CoV-2/SH01/human/2020/CHN (GenBank accession number MT121215), and ORFs were predicted against the formatted database using Prokka (-gcode 1) (68). Further, the GC content information was generated using the QUAST standalone tool (69).

### Phylogenetic analysis.

To infer the phylogeny, the core gene alignment was generated using MAFFT (70) within the Roary Package (71). Further, the phylogeny was inferred using the maximum likelihood method and the Tamura-Nei model (72) at the 1,000-bootstrap level in MEGAX (73) and was visualized in interactive Tree of Life (iTOL) (74) and GrapeTree (75).

We also constructed the gene phylogeny using nucleocapsid and spike proteins of 95 SARS-CoV-2 strains along with members of the *Coronaviridae* family present in synanthropic animals. The multiple-sequence alignment was performed using the MUSCLE (76) aligner, and the phylogeny was constructed using the neighbor joining method and MEGAX software (73). Further, the trees were visualized in interactive Tree of Life (iTOL) (74).

### Single nucleotide polymorphism and structural analysis.

To determine each single nucleotide polymorphism (SNP), whole-genome alignments were made using the libMUSCLE aligner. For this, we used annotated GenBank of SARS-CoV-2/SH01/human/2020/CHN (GenBank accession no. MT121215) as the reference in the parsnp tool of Harvest suite (77). As only genomes within a specified MUMI distance threshold are recruited, we used option -c to force inclusion of all the strains. For the output, it produced a core-genome alignment, variant calls, and a phylogeny based on single nucleotide polymorphisms. The SNPs were further visualized in Gingr, a dynamic visual platform (77). Further, the tree was visualized in interactive Tree of Life (iTOL) (74).

The 3D structures for nsp13 and nsp16 were predicted using the amino acid sequence from the wild-type reference (MT121215) and mutants MT163719 and MT188341, respectively. The 3D structures were predicted using the ExPASy Web interface tool, the SwissModel server homology modeling pipeline (78). The structures were subjected to energy minimization using UCSF Chimera v.1.13.1 software (79). The predicted models were subjected to validation using a Ramachandran plot of the proteins and the structure assessment tool in SWISS-MODEL. The structures were compared to assess the effect of mutation, and the levels of stability were compared using the HOPE (80), iPBA (81), and I-mutant v3.0 (82) servers. The secondary protein structure motifs were created by PDB-sum using v3.0 of Gail Hutchinson’s PROMOTIF program (83, 84) and compared for changes. The active sites and motifs were predicted for both models by the use of the PROSITE online server of ExPasy (https://prosite.expasy.org/). The 3D structures of the predicted models were visualized using UCSF Chimera software v.1.13.1 (79).

### SARS-CoV-2 protein annotation and host-pathogen interactions.

The SARS-CoV-2/SH01/human/2020/CHN virus genome having accession no. MT121215.1 was used for protein-protein network analysis. The data corresponding to the interaction of SARS-COV-2 and human host were extracted from the IntAct database with high-confidence values ranging between 0.74 and 0.97 (MIscore) (34–36). The network was visualized using Cytoscape v3.7.2 (85) and was analyzed to gain insights into the network topology using Network Analyzer, a plugin of Cytoscape. Network topology results gave an overview of network topological features, including diameter, degree distribution, shortest path distribution, and clustering coefficient of the interaction network. The network was investigated with the power law equation, and degree-based analyses were carried out. Further, the human proteins interacting with individual viral proteins were subjected to functional annotation. Gene ontology (GO) analysis was performed using ClueGo (86), selecting the Kyoto Encyclopedia of Genes and Genomes (KEGG) (87, 88), Gene Ontology—biological function database, and Reactome Pathways (89) databases. The ClueGo parameters were as follows: Go Term Fusion selected; pathways or terms of the associated genes, ranked based on the *P* value corrected with Bonferroni stepdown (*P* values of <0.05); GO tree interval, all levels; GO term minimum number of genes, 3; threshold, 4% of genes per pathway; kappa score, 0.42. The GO term value was reduced to 1 gene for the S and E proteins. Gene ontology terms are presented as nodes and clustered together based on the similarity of genes corresponding to each term or pathway.

### Analysis of natural selection.

To determine the evolutionary pressure on viral proteins, *dN*/*dS* values were calculated for 9 ORFs of all strains. The orthologous gene clusters were aligned using MUSCLE v3.8 (32) and further processed for removal of stop codons using HyPhy v2.2.4 (90). The single-likelihood ancestor counting (SLAC) method was used in Datamonkey v2.0 (91) (http://www.datamonkey.org/slac) to calculate the *dN*/*dS* value for each orthologous gene cluster. The *dN*/*dS* values were plotted in R (R Development Core Team, 2015).
