# Supplementary figures and images for: Comparative Genomic Analysis of Rapidly Evolving SARS-CoV-2 Reveals Mosaic Pattern of Phylogeographical Distribution
Source: mSystems. 2020 Jul 28;5(4):e00505-20. doi: 10.1128/mSystems.00505-20 (PMC7394360; doi:10.1128/mSystems.00505-20)

(A)

Tree scale: 0.1

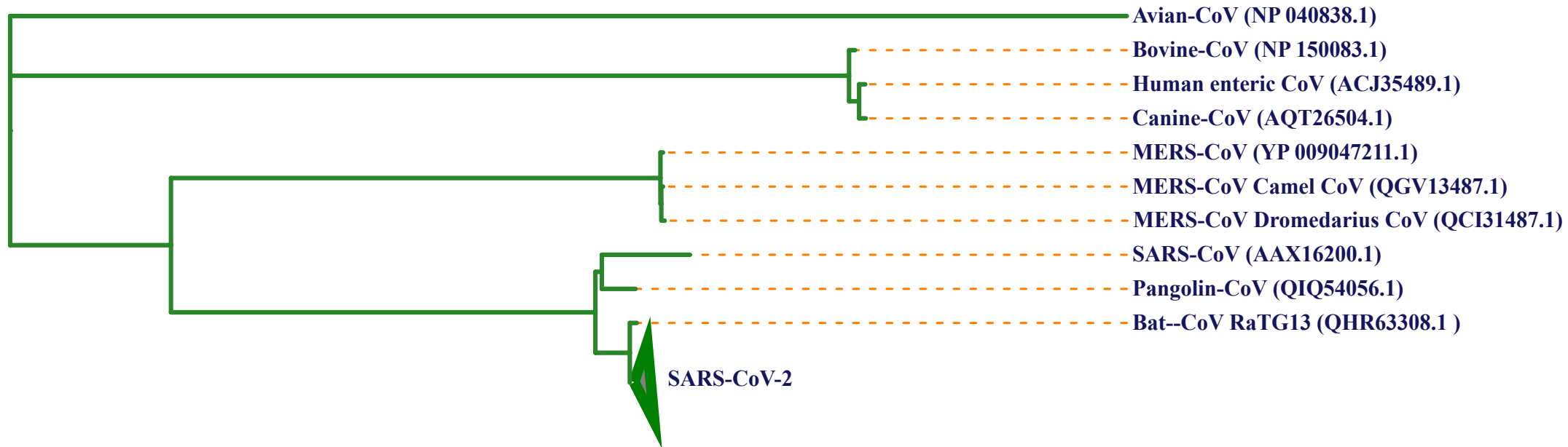

(B)

Tree scale: 0.01

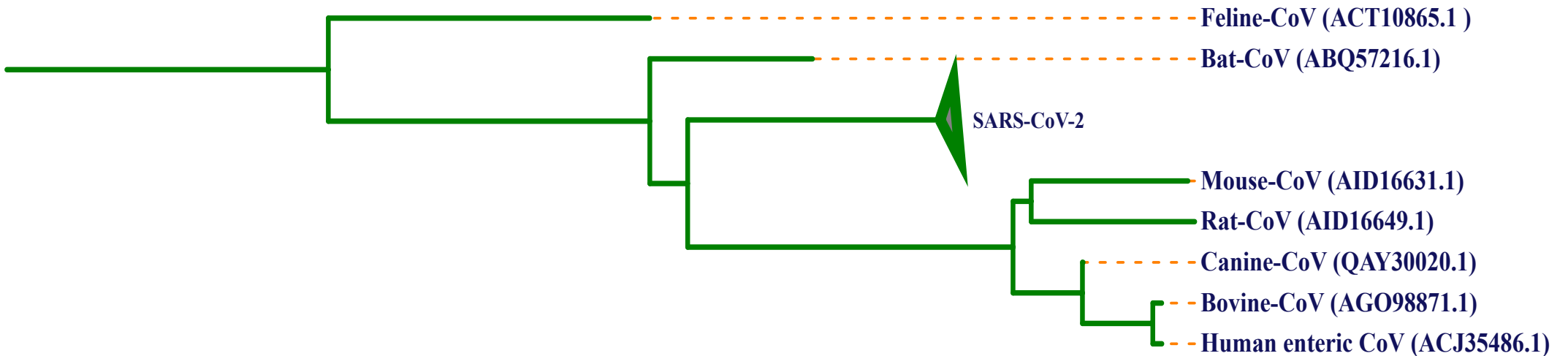

Supplement: FIG S1 [file mSystems.00505-20-sf001.pdf]
