# Supplementary material for: Comparative Genomic Analysis of Rapidly Evolving SARS-CoV-2 Reveals Mosaic Pattern of Phylogeographical Distribution
Source: mSystems. 2020 Jul 28;5(4):e00505-20. doi: 10.1128/mSystems.00505-20 (PMC7394360; doi:10.1128/mSystems.00505-20)
Supplement: TABLE S1 [file mSystems.00505-20-st001.docx]

| **S. No.** | **Strain** | **Mutation** |
| --- | --- | --- |
| 1. | MT027064 | H49Y |
| 2. | MT049951 | Y28N |
| 3. | MT012098 | DELETION OF ONE AMINO ACID AND FRAMESHIT AT POSITION 145 |
| 4. | MT039890 | S221W |
| 5. | MT159716 | F157L |
| 6. | MT1849210 | G181V |
| 7. | MT007544 | S247R |
| 8. | MT163720 | H655Y |
| 9. | MT093571 | F797C |
| 10. | MN988713 | N824X |
| 11. | MT050493 | A930V |
